# Earlier snowmelt and warming lead to earlier but not necessarily more plant growth

**DOI:** 10.1093/aobpla/plw021

**Published:** 2016-04-13

**Authors:** Carolyn Livensperger, Heidi Steltzer, Anthony Darrouzet-Nardi, Patrick F. Sullivan, Matthew Wallenstein, Michael N. Weintraub

**Affiliations:** 1 Department of Ecosystem Science and Sustainability, Colorado State University, Fort Collins, CO 80523, USA; 2 Biology Department, Fort Lewis College, Durango, CO 81301, USA; 3 Department of Biological Sciences, University of Texas at El Paso, El Paso, TX 79968, USA; 4 Environment and Natural Resources Institute, University of Alaska Anchorage, Anchorage, AK 99508, USA; 5 Department of Environmental Sciences, University of Toledo, Toledo, OH 43606, USA

**Keywords:** Arctic tussock tundra, climate change, plant phenology, plant production, seasonality

## Abstract

Climate change over the past ∼50 years has resulted in earlier occurrence of plant life-cycle events for many species. Across temperate, boreal and polar latitudes, earlier seasonal warming is considered the key mechanism leading to earlier leaf expansion and growth. Yet, in seasonally snow-covered ecosystems, the timing of spring plant growth may also be cued by snowmelt, which may occur earlier in a warmer climate. Multiple environmental cues protect plants from growing too early, but to understand how climate change will alter the timing and magnitude of plant growth, experiments need to independently manipulate temperature and snowmelt. Here, we demonstrate that altered seasonality through experimental warming and earlier snowmelt led to earlier plant growth, but the aboveground production response varied among plant functional groups. Earlier snowmelt without warming led to early leaf emergence, but often slowed the rate of leaf expansion and had limited effects on aboveground production. Experimental warming alone had small and inconsistent effects on aboveground phenology, while the effect of the combined treatment resembled that of early snowmelt alone. Experimental warming led to greater aboveground production among the graminoids, limited changes among deciduous shrubs and decreased production in one of the dominant evergreen shrubs. As a result, we predict that early onset of the growing season may favour early growing plant species, even those that do not shift the timing of leaf expansion.

## Abstract (precis)

In the Arctic, rapid warming due to climate change has led to earlier springs and increased plant production at a landscape scale. However, individual species in the tundra may respond differently to growth cues like timing of snowmelt and temperature. While many tundra species expand their leaves earlier due to early snowmelt and warming, this does not always lead to increased production. In our study, early growing species such as cottongrass (*Eriophorum vaginatum*) increased production under early snowmelt and warmed conditions, while later growing species did not. Early onset of the growing season may favor these early growing species.

## Introduction

Seasonality in temperate to polar ecosystems is shifting through earlier seasonal warming and changes in precipitation regimes that lead to earlier snowmelt (Schwartz *et al*. 2006; Hayhoe *et al*. 2007; Ernakovich *et al*. 2014). Plant communities are responding through changes in timing of life-cycle events such as leaf expansion and flowering (i.e. phenology) (Fitter and Fitter 2002; Thompson and Clark 2008), shifts in species relative abundance (Harte and Shaw 1995; Willis *et al*. 2008), species’ range shifts (Walther *et al*. 2005; Gottfried *et al*. 2012) and greater aboveground plant production (Knapp and Smith 2001; Wang *et al*. 2012). These observations of altered seasonality and plant community changes correspond to a period of increasing global temperatures, but experiments are still needed to determine mechanisms and develop predictive models as climate change continues (Pau *et al*. 2011; Richardson *et al*. 2012).

Temperature and photoperiod are known plant phenological cues that determine the timing of spring events, such as bud burst, leaf emergence and canopy development, and flowering (Cleland *et al*. 2007; Körner and Basler 2010; Polgar and Primack 2011). Experimental warming studies using different techniques, such as active warming through overhead infrared heaters or passive warming with open-top chambers (OTCs), demonstrate that many species begin growth and flowering earlier in warmed versus control plots (Cleland *et al*. 2006; Sherry *et al*. 2007; Reyes-Fox *et al*. 2014). However, responses can vary with some species not shifting or delaying the timing of spring events under warmed conditions (Hollister *et al*. 2005*a*; Reyes-Fox *et al*. 2014; Marchin *et al*. 2015). Similarly, long-term observations of phenological response to climate warming over time show an overall advance in timing of spring of an estimated 5–6 days °C^−1^ (Wolkovich *et al*. 2012), but with interspecific variation (Fitter and Fitter 2002; Menzel *et al*. 2006). The variation in response suggests that phenology is cued by other environmental variables (e.g. photoperiod) for species within and across diverse plant communities from tundra, grassland and forest biomes.

The influence of snow cover on plant phenology is less well understood, in part because temperature change due to climate change and experimental warming influence when an area becomes snow-free. Development of early emerging species may be closely synchronized with timing of snowmelt in Arctic and alpine ecosystems (Galen and Stanton 1995; Høye *et al*. 2007), and indeed, later snowmelt due to increased snow depth has been shown to delay bud break of deciduous shrubs (Borner *et al*. 2008; Sweet *et al*. 2014). However, few experiments have examined the isolated effects of early snowmelt and summer warming in the Arctic or alpine, and often these effects are confounded either by the use of warming treatments such as OTCs to accelerate snowmelt or by the snow removal that reduces water inputs (Wipf and Rixen 2010).

Multifactor global change experiments have shown that plant production is sensitive to manipulations of abiotic factors, including air and soil warming, nutrients, CO_2_ and precipitation (Fay *et al*. 2003; Zavaleta *et al*. 2003; Dukes *et al*. 2005; Dawes *et al*. 2011). Response to these factors is complex, with variation across plant communities due to differences in limiting factors (Smith *et al*. 2015), and variation within communities due to differences in functional group responses (Zavaleta *et al*. 2003; Wahren *et al*. 2005; Muldavin *et al*. 2008). In the Arctic, production is strongly limited by nutrient availability, which in turn is sensitive to temperature and ongoing changes in the timing of seasonal climatic events such as snowmelt, soil thaw, the onset of freezing and snowfall (Billings and Mooney 1968; Weintraub and Schimel 2005). In recent years, both observational and experimental studies have linked increased production, specifically that of deciduous shrubs and graminoids, to warmer temperatures (Tape *et al*. 2006; Walker *et al*. 2006; Forbes *et al*. 2010; Elmendorf *et al*. 2012*b*; Sistla *et al.* 2013). However, a number of experiments that have manipulated summer temperature in both Arctic and alpine regions did not find a consistent increase in community-level aboveground net primary production (ANPP); rather, individual species or functional groups varied in their response (Chapin *et al*. 1995; Harte and Shaw 1995; Hollister *et al*. 2005*b*). Evergreen shrubs have responded to warming with positive, negative or no changes in production (Hollister *et al*. 2005*b*; Walker *et al.* 2006; Campioli *et al*. 2012), and they may be less likely to show short-term growth responses due to their conservative growth strategy (Chapin and Shaver 1996; Starr *et al*. 2008).

Changes in plant production may also be expected to vary in relation to changes in the timing of growth; for example, earlier leaf expansion may lead to greater productivity (Richardson *et al*. 2010). There is evidence that phenological ‘tracking’ of climate change across biomes can result in positive growth responses, through increased abundance, production or flowering effort (Cleland *et al*. 2012). However, physiological constraints and interactions of the affected species may prevent some plants from taking advantage of an earlier start to the growing season (Schwartz 1998; Richardson *et al*. 2010; Polgar and Primack 2011). One such constraint could be negative impacts of exposure to cold temperatures and freezing damage if snow melts early (Inouye 2008; Wipf *et al*. 2009). Differences in the onset and duration of plant growth can also vary due to differences in plant community composition; for example, deciduous shrub-dominated communities in Arctic tundra were shown to have longer peak growing seasons and greater carbon uptake than evergreen/graminoid communities in the same region (Sweet *et al*. 2015).

In the Arctic, climate is changing at a faster rate than in other regions, a trend that is expected to continue (Christensen *et al*. 2013). Rapidly increasing air temperature (∼1 °C decade^−1^) (Christensen *et al*. 2013), earlier snowmelt (3–5 days decade^−1^) and later snowfall are changing the seasonality of this ecosystem (Serreze *et al*. 2000; Ernakovich *et al*. 2014). Landscape-scale observations via remote sensing suggest that vegetation phenology in the Arctic is indeed advancing and plant production is increasing (Myneni *et al*. 1997; Jia *et al*. 2003, 2009; Fraser *et al*. 2011; Zeng *et al*. 2011). Earlier snowmelt, especially in combination with warmer temperatures in early spring, should benefit plant growth, since it is the time of year with the greatest light and nutrient availability (Weintraub and Schimel 2005; Edwards and Jefferies 2013). However, experiments are needed to determine how shifts in seasonality will affect phenology of Arctic species, and how changes in phenology affect plant productivity and future community composition.

In Arctic tussock tundra, we established a 3-year study in which we altered seasonality through the independent and combined manipulation of air temperature and timing of snowmelt. We examined the response of spring phenology and plant production for key tundra species and hypothesized that:

1. The timing of snowmelt and temperature are cues for initiating plant growth. We predicted that leaf appearance and expansion would advance due to early snowmelt and air warming for all species.
2. The timing of snowmelt and temperature affect plant production. We predicted that early snowmelt and warmer temperatures would increase production of deciduous shrub, graminoid and forb species, but would not change production of evergreen shrubs.
3. The timing of plant growth affects plant production. We predicted that earlier leaf expansion would lead to greater aboveground biomass at peak season.

## Methods

### Site description

The experiment was conducted near Imnavait Creek on the North Slope of Alaska, close to the Arctic Long-Term Ecological Research (LTER) site at Toolik Field Station. The plant community at Imnavait is moist acidic tussock tundra, characterized by the tussock forming sedge *Eriophorum vaginatum* and a high moss cover, including *Hylocomium* spp., *Aulacomnuim* spp. and *Dicranum* spp. Associated species include another sedge, *Carex bigelowii*, the deciduous shrubs *Betula nana* and *Salix pulchra*, the evergreen shrubs *Ledum palustre*, *Vaccinium vitis-idaea* and *Cassiope tetragonum*, and a variety of forbs **[see Supporting Information****—Table S1****]**. The old (∼120 000–600 000 years; Whittinghill and Hobbie 2011), acidic soil (mean pH of 4.5) at this site is underlain by continuous permafrost, with an uneven surface layer of organic material 0–20 cm thick (Walker *et al*. 1994) and variable soil moisture.

### Altered seasonality

For 3 years (2010–12), snowmelt was accelerated in five 8 × 12 m plots using radiation-absorbing black 50 % shade cloth that was placed over the snowpack in late April–early May. The dark fabric accelerated melt and allowed for minimal disturbance of the snowpack. The fabric was removed when plots became 80 % snow-free (determined by daily visual estimates). In 2012, we achieved a 10-day acceleration in the timing of snowmelt with early snowmelt plots becoming snow-free on May 16 and control plots snow-free on May 26. Snow was melted 4 and 15 days earlier in 2010 and 2011, respectively. As plots became snow-free, (OTCs were deployed on subplots within the accelerated snowmelt and control areas. The OTCs are hexagonal chambers with sloping sides, constructed of Plexiglas material that allows transmittance of wavelengths of light in the visible spectrum, enabling passive warming primarily through trapping solar radiation (Marion *et al*. 1997). Open-top chambers warmed air temperatures by an average of 1.4 °C in 2012. Further details of treatment effects on air temperature, soil temperature and soil moisture are available in **Supporting Information—Table S2**. The approximate area of both control and warming subplots was 1 m^2^. Treatments were replicated five times in a full factorial, randomized split-plot design. Treatment abbreviations are as follows throughout the article: control (C), warming (W), early snowmelt (ES) and combined (W × ES).

### Phenology

Five individuals of seven species were marked in each subplot and phenology events were monitored every 2–3 days from snowmelt through mid-August. Observations of ‘leaf appearance’ and ‘leaf expansion’ were recorded for each individual. Although definitions of events varied between functional groups, we generally considered leaf appearance to be the first observation of new green leaves and leaf expansion to be when an individual had a leaf that was fully expanded or had reached a previously determined size. For deciduous shrubs (*B. nana* and *S. pulchra*), leaf appearance was recorded at the first observed leaf bud burst, and leaf expansion when an individual had at least one fully unfurled leaf anywhere on the plant. Similarly, evergreen shrub (*L. palustre* and *V. vitis-idaea*) leaf appearance was recorded when the first leaf bud was visible, and full leaf expansion occurred when at least one leaf bud was fully open and leaves unfurled. *Eriophorum vaginatum* retains green leaf material over winter and often begins growth of new leaves and re-greening of old leaves before snow is completely melted (Chapin *et al*. 1979). Therefore, we recorded leaf appearance (new leaves >1 cm length) for *E. vaginatum* on the day of snowmelt, but did not consider this as a treatment effect. Rather than continuously measuring leaf length to record full leaf expansion, we determined leaf expansion for *E. vaginatum* to have occurred when a new leaf reached >4 cm length. We only considered growth of new leaves, which were identified as those with no senescent material at the leaf tip. We followed similar protocol for *C. bigelowii* leaf appearance (new leaf >1 cm length) and leaf expansion (new leaf >4 cm length), but leaf appearance was considered a treatment effect. First leaf appearance for the forb *P. bistorta* was marked when leaves were visible (generally >1 cm length) and leaf expansion when leaves were fully unrolled and >5 cm length.

### Plant production

A destructive harvest to measure plant production, as characterized by growth of individuals in the current year, was carried out on the same species for which phenology was observed. The seven species chosen represented four functional groups and comprised the majority of vascular plant cover at our site **[see Supporting Information—Table S1]**. The harvest took place in the third year of treatments at peak growing season, which was determined by phenology observations and analysis of daily normalized difference vegetation index measurements showing that peak greenness (i.e. full canopy development) had occurred in each treatment (C. Livensperger and H. Steltzer, unpublished data). Randomly selected individuals were clipped in the field, and then taken back to the laboratory where old and new growth was separated and biomass measured. Eight individuals each of *B. nana*, *S. pulchra* and *L. palustre*, and 16 individuals each of *V. vitis-idea*, *E. vaginatum*, *C. bigelowii* and *P. bistorta* were collected from each subplot and pooled by species. Plant material was separated by tissue type, dried at 60 °C for 48 h and weighed.

Mean individual production for each species was calculated as the sum of current years’ biomass divided by the number of individuals collected. Current years’ biomass included leaves, new stems and secondary growth for shrub species. For graminoids and a forb, all live aboveground plant tissue was used, which may have included some growth from previous years for *E. vaginatum* and *C. bigelowii*. We calculated current annual secondary stem growth for *B. nana*, *S. pulchra* and *L. palustre* as a proportion of standing stem biomass, using previously determined annual growth rates of woody stems from the nearby Toolik Lake LTER site (Bret-Harte *et al*. 2002). For these species, leaves contributed more to total biomass than the calculated secondary growth. Secondary growth for the remaining shrub species, *V. vitis-idaea*, is negligible and, therefore, was left out of production calculation for this species (Shaver 1986). Standing stem biomass, excluding current seasonal growth, for individual shrub stems varied among plots and likely is a result of variation prior to when the experiment was established. To control for this variation and better detect treatment effects, individual production data are presented in relation to standing stem biomass excluding current annual growth (i.e. g new production/g standing stem biomass).

### Statistical analyses

For all analyses, the experiment was treated as a blocked split-plot design, where a large early snowmelt plot paired with an equally sized control plot comprise a single block. Plant responses and environmental variables were analysed using a mixed-model analysis of variance (ANOVA; SAS v 9.2, SAS Institute, Inc., Cary, NC, USA), with early snowmelt (ES) as the main plot factor and warming (W) as the within plot factor. A random effect of block was included to control for inherent variation between the five replicates. All data were checked for normality and were found to meet the assumptions of ANOVA. Linear regression was used to analyse the relationship between phenology and plant growth.

## Results

### Plant phenology

**Figure 1.**
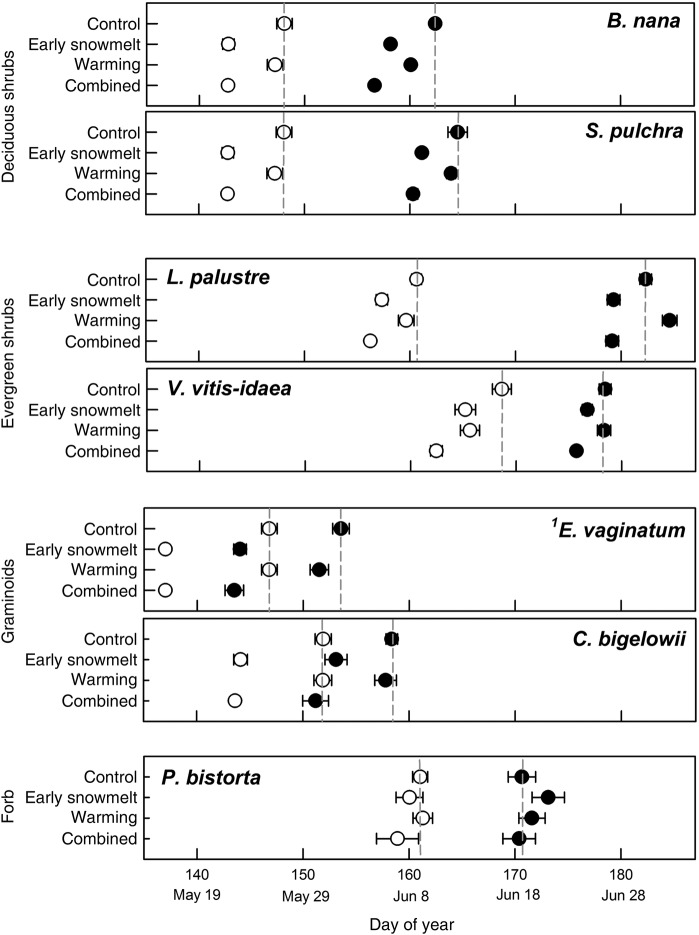
Dates of early-season phenology events, where open circles represent leaf appearance and filled circles represent leaf expansion. Points are the mean date of event ± 1 SEM. Vertical dashed lines denote mean event date for control plots. ^1^*E. vaginatum* initiates growth underneath the snowpack so we did not consider leaf appearance to be a treatment effect; however, the event date is shown to signify the presence of new leaves at the time of snowmelt.

**Figure 2.**
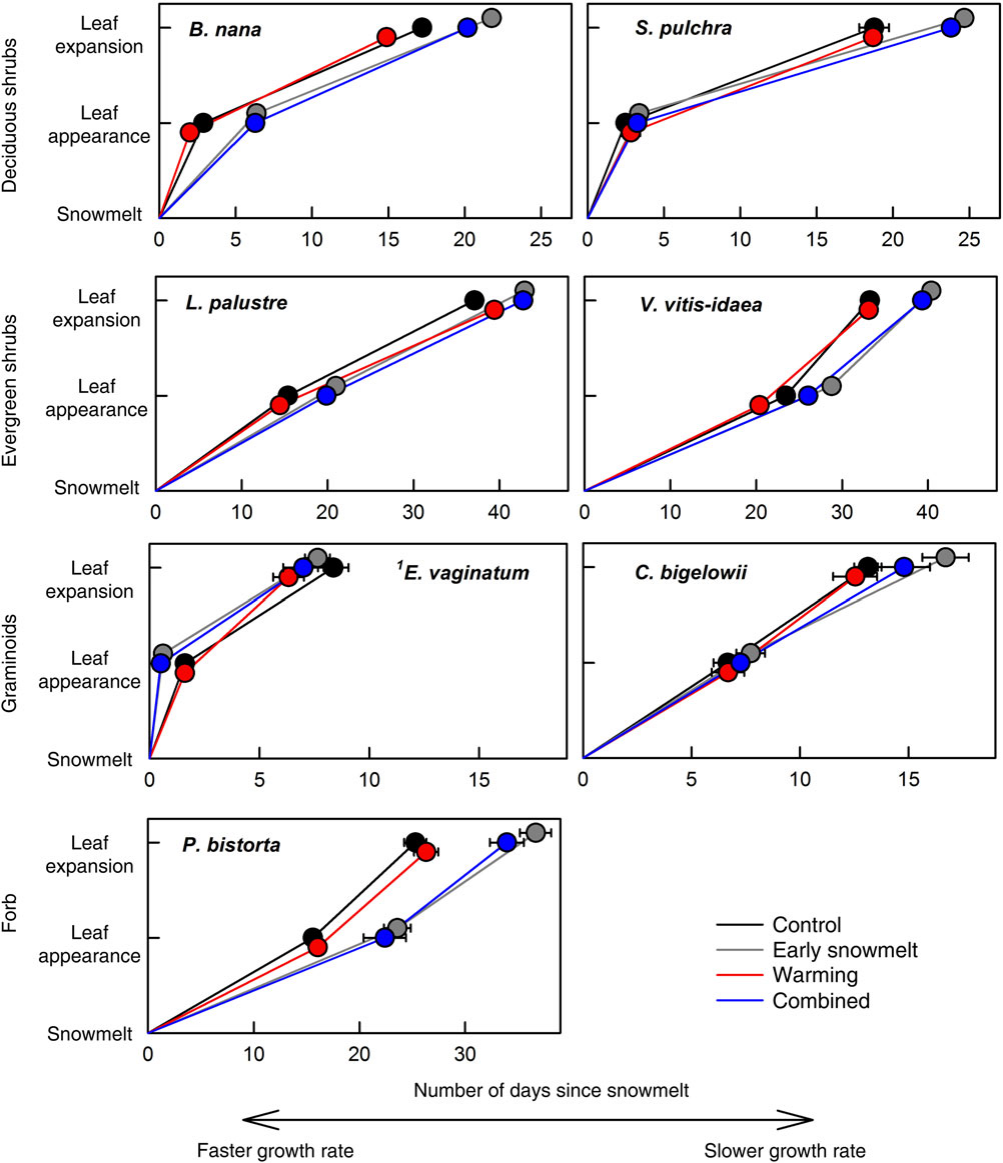
Number of days since snowmelt for early-season phenology events. Points are average number of days since snowmelt ± 1 SEM. A greater number of days until full leaf expansion are interpreted as a slower growth rate. Note different scales on *x*-axes. ^1^As noted in Fig. 1, leaf appearance of *E. vaginatum* is not considered a treatment effect but is shown for clarity.

Early snowmelt was a strong driver of change in both the timing and rate of leaf appearance and expansion. These events advanced due to early snowmelt alone for all species except the forb, *P. bistorta* (Fig. 1), and the amount of change in timing varied between events, species and functional groups. The largest change in timing was a 10-day advance in leaf expansion for *E. vaginatum* (Fig. 1), corresponding to the 10-day advance in snowmelt through our snow manipulation. Leaf appearance and expansion of evergreen and deciduous shrubs were significantly earlier due to early snowmelt alone, advancing by 1–8 days for *B. nana*, *S. pulchra*, *L. palustre* and *V. vitis-idaea* (Fig. 1, Table 1). The advancement of leaf appearance versus leaf expansion differed in magnitude for *S. pulchra*, *V. vitis-idaea* and *C. bigelowii*, by increasing the number of days between leaf appearance and leaf expansion by 2–5 days. For example, in the early snowmelt treatment, leaf appearance for *S. pulchra* occurred 8 days earlier than the control, while leaf expansion advanced by only 3 days. For deciduous shrubs, evergreen shrubs and the forb, the shift in phenology was less than the 10-day advance in snowmelt, increasing the number of days after snowmelt to when canopy development (i.e. leaf expansion) began; this effectively slowed the rate of plant production (Fig. 2, Table 2). The sedges, *E. vaginatum* and *C. bigelowii*, did not follow this pattern, with no evidence of a change in the number of days between leaf appearance and expansion (Fig. 2).

**Table 1.** Results of mixed-model ANOVA on timing of early-season phenology events. Leaf appearance for *E. vaginatum* was not considered a treatment effect and was excluded from the analysis. Bold values indicate a significant main effect of the treatment at *P* ≤ 0.05.

	Warming			Early snowmelt			Warming × Early snowmelt		
	df	*F*	*P*	df	*F*	*P*	df	*F*	*P*
Leaf appearance									
*B. nana*	1, 85	0.88	0.3503	1, 4	9.46	**0.0373**	1, 85	1.49	0.2253
*S. pulchra*	1, 71	0.21	0.6501	1, 8	20.24	**0.0019**	1, 71	0.15	0.7045
*L. palustre*	1, 86	5.75	**0.0186**	1, 4	21.53	**0.0099**	1, 86	0.00	0.9775
*V. vitis-idaea*	1, 83	15.08	**0.0002**	1, 4	8.92	**0.0405**	1, 83	0.01	0.9241
*C. bigelowii*	1, 83	0.16	0.6910	1, 4	65.58	**0.0012**	1, 83	0.20	0.6533
*P. bistorta*	1, 65	0.13	0.7212	1, 7	1.34	0.2829	1, 65	0.25	0.6184
Leaf expansion									
*B. nana*	1, 86	55.71	**<0.0001**	1, 4	79.28	**0.0008**	1, 86	2.33	0.1308
*S. pulchra*	1, 77	1.86	0.1766	1, 77	42.81	**<0.0001**	1, 77	0.04	0.8329
*L. palustre*	1, 86	3.26	0.0743	1, 8	30.97	**0.0006**	1, 86	3.95	0.0501
*V. vitis-idaea*	1, 88	1.59	0.2108	1, 88	19.73	**<0.0001**	1, 88	1.04	0.3100
*E. vaginatum*	1, 86	3.36	0.0702	1, 4	37.14	**0.0033**	1, 86	1.38	0.2436
*C. bigelowii*	1, 84	1.64	0.2037	1, 4	31.25	**0.0053**	1, 84	0.55	0.4599
*P. bistorta*	1, 70	0.13	0.7182	1, 70	0.30	0.5882	1, 70	1.33	0.2525

**Table 2.** Results of mixed-model ANOVA on duration of time since snowmelt for early-season phenology events. Leaf appearance for *E. vaginatum* was not considered a treatment effect and was excluded from the analysis. Bold values indicate a significant main effect of the treatment at *P* ≤ 0.05.

	Warming			Early snowmelt			Warming × Early snowmelt		
	df	*F*	*P*	df	*F*	*P*	df	*F*	*P*
Leaf appearance									
*B. nana*	1, 86	1.00	0.3210	1, 8	22.56	**0.0015**	1, 86	1.35	0.2479
*S. pulchra*	1, 74	0.14	0.7070	1, 7	1.25	0.2975	1, 74	0.26	0.6112
*L. palustre*	1, 86	5.73	**0.0188**	1, 4	33.32	**0.0047**	1, 86	0.00	0.9889
*V. vitis-idaea*	1, 83	15.03	**0.0002**	1, 4	30.73	**0.0057**	1, 83	0.01	0.9179
*C. bigelowii*	1, 87	0.16	0.6933	1, 87	1.81	0.1823	1, 87	0.19	0.6639
*P. bistorta*	1, 65	0.10	0.7576	1, 65	36.75	**<0.0001**	1, 65	0.49	0.4859
Leaf expansion									
*B. nana*	1, 85	55.48	**<0.0001**	1, 4	40.62	**0.0030**	1, 85	2.36	0.1281
*S. pulchra*	1, 75	0.89	0.3475	1, 4	45.21	**0.0028**	1, 75	0.35	0.5537
*L. palustre*	1, 90	3.44	0.0669	1, 90	59.34	**<0.0001**	1, 90	4.16	**0.0444**
*V. vitis-idaea*	1, 85	1.50	0.2234	1, 4	173.94	**0.0002**	1, 85	0.95	0.3323
*E. vaginatum*	1, 87	3.51	0.0645	1, 4	0.00	0.9497	1, 87	1.33	0.2526
*C. bigelowii*	1, 84	1.71	0.1951	1, 4	4.42	0.1025	1, 84	0.54	0.4635
*P. bistorta*	1, 70	0.26	0.6115	1, 70	51.99	**<0.0001**	1, 73	1.66	0.2013

Warming also advanced the timing of leaf appearance and expansion for most species, but to a lesser extent than early snowmelt (Fig. 1, Table 1). All of the deciduous shrub and graminoid species advanced leaf phenology with warming alone, but only by 1 or 2 days (Fig. 1, Table 1). Evergreen shrubs showed contrasting responses to warming: leaf appearance for *V. vitis-idaea* advanced by 3 days, while *L. palustre* leaf expansion was delayed for 2 days (Fig. 1, Table 1). Warming generally did not alter phenology in relation to the timing of snowmelt (Fig. 2, Table 2). One exception is that warming led to significantly faster leaf expansion following snowmelt for *B. nana*, effectively speeding plant production.

Phenological responses to the combination of early snowmelt and warming were generally comparable with the response to early snowmelt alone (Figs 1 and 2), and the interactive effect of warming × early snowmelt on phenology was never significant (Table 1). For evergreen shrubs, leaf appearance occurred earliest with the combined treatment, which was 1–3 days earlier than in snowmelt and warming alone (Fig. 1).

### Plant production

Although phenological events often occurred earlier in the year due to earlier snowmelt and warming, an increase in individual production was rarely observed. Rather, responses to early snowmelt and warming varied within and among functional groups. Differences were rarely significant (Table 3), in part due to the challenge of quantifying plant production in an ecosystem with high spatial variation.

**Table 3.** Results of mixed-model ANOVA on individual biomass. Bold values indicate a significant main effect of the treatment at *P* ≤ 0.05.

	Warming			Early snowmelt			Warming × Early snowmelt		
	df	*F*	*P*	df	*F*	*P*	df	*F*	*P*
Individual biomass									
*B. nana*	1, 16	0	0.9859	1, 16	0.22	0.6466	1, 16	1.02	0.3287
*S. pulchra*	1, 12	0.17	0.6900	1, 12	0.18	0.6783	1, 12	1.18	0.2990
*L. palustre*	1, 8	0.73	0.4185	1, 8	1.35	0.2782	1, 8	0.43	0.5283
*V. vitis-idaea*	1, 16	**5.39**	**0.0338**	1, 16	0.19	0.6686	1, 16	0.95	0.3454
*E. vaginatum*	1, 8	**7.48**	**0.0256**	1, 4	0.20	0.6757	1, 8	2.35	0.1635
*C. bigelowii*	1, 12	1.51	0.2425	1, 12	1.04	0.3277	1, 12	0.16	0.6926
*P. bistorta*	1, 16	0.85	0.3831	1, 16	0.08	0.7808	1, 16	0.28	0.6106

However, the magnitude of change often represented a high proportion of production in this low productivity system. Deciduous shrub species differed in their response, with *S. pulchra* decreasing individual production by 6–11 % and *B. nana* showing little change across the three treatments (Table 3). Evergreen shrub species increased individual production by 28 and 8 % for *L. palustre* and *V. vitis-idaea*, respectively, due to early snowmelt (Table 3). Individual production of *P. bistorta*, the forb, was highly variable within treatments; for example, control plants ranged from 36 to 297 mg biomass. The most evident response for this species was a large, but non-significant, decrease (36 %) in production due to early snowmelt (Table 3).

**Figure 3.**
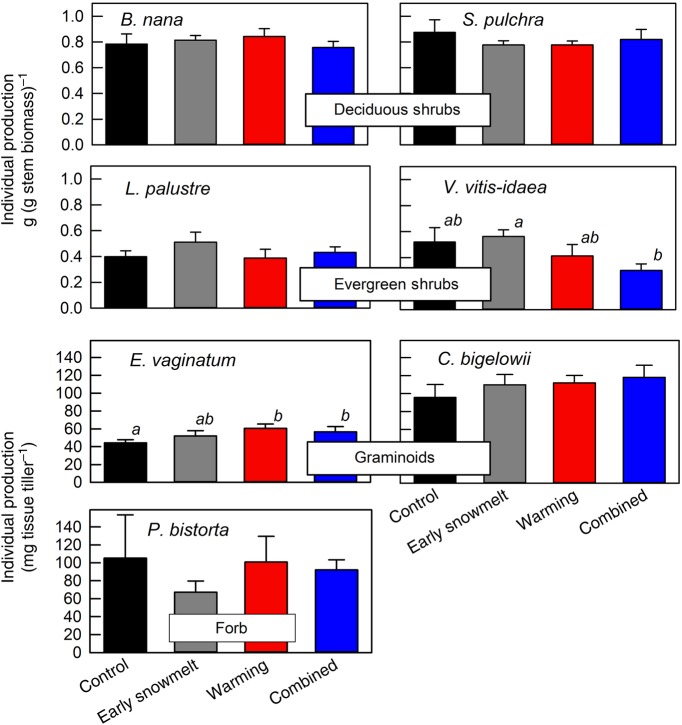
Biomass of individual species harvested in the third year of altered seasonality. For deciduous and evergreen shrubs, bars represent means of proportion of current annual growth to standing stem biomass of the individual, ±1 SEM. For graminoids and a forb, bars represent means of aboveground biomass, ±1 SEM. Letters (a and b) represent groupings based on least squares means of the ANOVA mixed model, where bars with the same letter are not statistically different at *P* ≤ 0.05.

The effect of warming on production was statistically significant for two species and led to the largest proportional changes (Fig. 3, Table 3). Both graminoid species responded positively to warming. Mean individual production for *E. vaginatum* increased by 36 %, which was the greatest proportional increase of any species (Fig. 3, Table 3). When early snowmelt and warming were combined, *E. vaginatum* increased individual production by 27 % relative to the control (Fig. 3, Table 3). The other graminoid, *C. bigelowii*, increased individual production by 17 % with warming and 24 % with warming and early snowmelt, although these increases were not significant (Fig. 3, Table 3). An evergreen shrub, *V. vitis-idaea*, had relatively large decreases relative to the control with warming (21 %) and the combined treatment (42 %), and the main effect of warming was significant (Table 3).

### Phenology and production relationship

**Figure 4.**
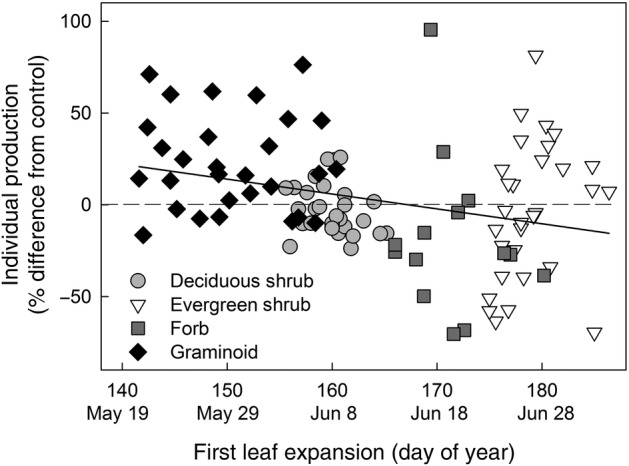
Relationship between phenology and production, with production (*y*-axis) represented as the per cent difference from the control mean ANPP for each species (100 × treatment − control mean/control). Each point represents one species, treatment and plot. The solid line is the linear regression (see Results for details).

**Figure 5.**
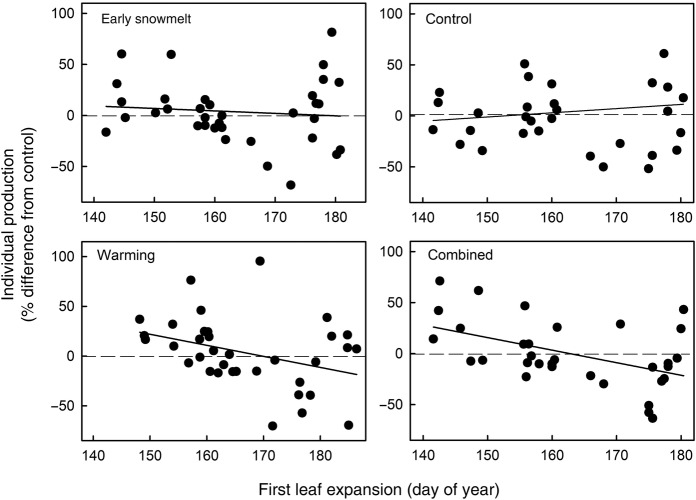
Relationship between phenology and production by treatment type. Data follow Fig. 4. Solid lines are linear regressions for each functional group (see Results for details).

**Figure 6.**
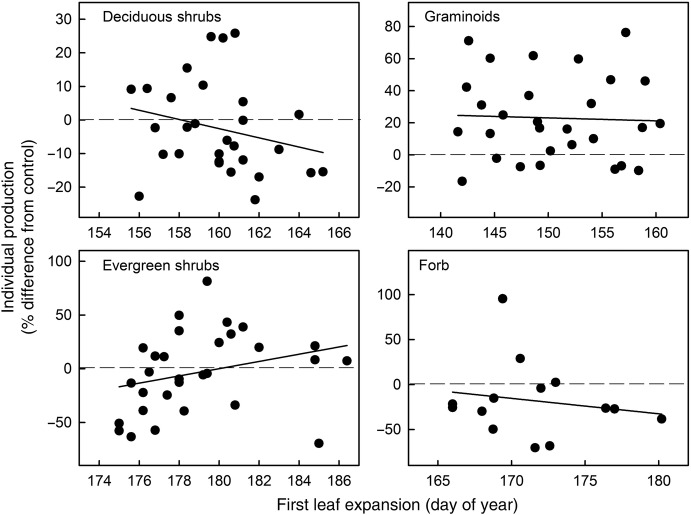
Relationship between phenology and production by functional group. Data follow Fig. 4. Solid lines are linear regressions for each functional group (see Results for details).

Across all species and for all treatments, earlier leaf expansion was associated with increased production (Fig. 4, *y* = 135.52–0.81*x*, *R*^2^ = 0.09, *P* = 0.0021). This relationship reflects differences in the timing of leaf expansion among growth forms and the response of individual plant production to early snowmelt and warming. Species varied in the timing of leaf expansion by 40 days, a range that was expanded by 14 days due to altered seasonality. Early expanding species (*E. vaginatum* and *C. bigelowii*) had increases in production, while later expanding species (*L. palustre* and *V. vitis-idaea*) had some increases and also large decreases in production as a result of warming. Across functional groups, warming drove the relationship between timing of leaf expansion and individual production, as shown by significantly negative regression slopes within the warming and combined treatments (Fig. 5; C: *y* = −62.7 + 0.41*x*, *R*^2^ = 0.01, *P* = 0.53; ES: *y* = 43.16–0.24*x*, *R*^2^ = 0.01, *P* = 0.547; W: *y* = 188.66–1.11*x*, *R*^2^ = 0.13, *P* = 0.03; W × ES: *y* = 200.26–1.23*x*, *R*^2^ = 0.24, *P* = 0.005). Within functional groups, there was no relationship between the timing of leaf expansion and individual production, despite earlier leaf expansion due to early snowmelt and warming (Fig. 6; deciduous shrubs: *y* = 216.43–1.37*x*, *R*^2^ = 0.06, *P* = 0.202; evergreen shrubs: *y* = −606.13 + 3.37*x*, *R*^2^ = 0.08, *P* = 0.129; graminoids: *y* = 50.74–0.18*x*, *R*^2^ = 0.002, *P* = 0.7; forb: *y* = 276.82–1.72*x*, *R*^2^ = 0.03, *P* = 0.582).

## Discussion

Our results showed consistent advancement of leaf appearance and expansion, indicating that spring phenology of moist acidic tundra species is sensitive to early snowmelt and warming, which is consistent with our first hypothesis. Warmer temperatures have been shown to advance spring phenology in other systems, particularly for deciduous shrubs where budburst is well predicted by growing degree-days (Polgar and Primack 2011). However, we found that species were more responsive to early snowmelt, by advancing timing of events to a greater extent than with warming alone. Timing of snowmelt has been shown to be a cue for spring phenology in Arctic and alpine ecosystems (Arft *et al*. 1999; Steltzer *et al*. 2009), but experiments often confound the effects of warming and timing of snowmelt. Arctic species generally have a wide range of physiological tolerance, allowing spring growth to occur despite temperatures at or near freezing (Billings and Mooney 1968), and our experiment shows that early snowmelt can advance phenology independent of warming.

Along with clear advances in spring phenology, we also observed slower rates of leaf expansion for many species in response to early snowmelt, supporting the conservative growth strategy demonstrated by many Arctic and alpine species to compensate for interannual variation in snowmelt timing (Billings and Mooney 1968). Surprisingly, only one species, *B. nana*, expanded its leaves at a faster rate with warming. It may be that soil temperature, which warmed to a lesser extent than air temperature **[see Supporting Information—Table S2]**, is an additional cue for rate of leaf expansion for most species. Plants that expand leaves early may be susceptible to frost damage if temperatures remain cold or freezing events occur (Inouye 2008; Wipf *et al*. 2009).

Production responses to warming and early snowmelt were dependent on growth form and individual species. One functional group (graminoids) matched our predicted direction of response, while others did not (forbs and deciduous and evergreen shrubs). Previous warming experiments have also shown interspecific variation within tundra communities, with graminoids and deciduous shrubs showing rapid change relative to evergreen shrubs and forbs (Chapin and Shaver 1985; Chapin *et al*. 1995; Hollister *et al*. 2005*b*). The response of graminoids in our study was consistent with these experiments, with both *E. vaginatum* and *C. bigelowii* increasing production in response to warming and early snowmelt. Although we measured biomass of individual tillers, new tiller recruitment is another likely mechanism by which either graminoid species could have increased biomass (Chapin and Shaver 1985). Graminoids were the only functional group that maintained their growth rates when snow was melted early, which may confer an advantage in accessing early-season nutrient pulses, and consequently increasing production in the same year (Shaver *et al*. 1986). Our results are generally consistent with past work on *E. vaginatum*, which showed that early-season air warming leads to accelerated leaf growth and earlier arrival at peak biomass (Sullivan and Welker 2005). Our observation that graminoids were also able to advance timing of early-season phenology to a greater extent than the other functional groups may be due to their ability to initiate growth underneath the snowpack and therefore have new leaves present at snowmelt in addition to green leaves that have overwintered (Chapin *et al*. 1979).

Warming resulted in a large decrease in production for the evergreen shrub, *V. vitis-idaea*, a species that has shown much variability in response to warming in previous experiments (Chapin *et al*. 1995; Arft *et al*. 1999; Zamin *et al*. 2014). A meta-analysis of warming experiments across the Arctic suggests that evergreen shrub response to warming depends on soil moisture regime, with plants in moist soils more often decreasing in abundance (Elmendorf *et al*. 2012*a*). Regardless, the large change in production that we observed was unexpected because evergreen shrubs have a conservative growth strategy, demonstrated by slower growth rates, lower specific leaf area and lower photosynthetic capacity than other species in the tundra community (Chapin and Shaver 1996; Starr *et al*. 2008). A decrease in new leaf biomass by *V. vitis-idaea* could be related to conditions in previous years, because evergreen shrub growth relies in part on nutrients stored in old leaves (Billings and Mooney 1968). Alternatively, *V. vitis-idaea* may be a poorer competitor than deciduous species (e.g. *B. nana*) for increased nutrients under warmed conditions (Shaver *et al*. 2001). Evergreen shrubs have the ability to access early-season nutrient pulses (McKane *et al*. 2002; Larsen *et al*. 2012) and photosynthesize under the snowpack (Starr and Oberbauer 2003), which may explain why both species increased production in response to early snowmelt, similar to graminoids. However, this does not explain why *V. vitis-idaea* would show the opposite response when early snowmelt was combined with warming.

Production of deciduous shrubs and a forb did not show clear responses to warming or early snowmelt. It may be that the 3-year duration of our study did not allow enough time for *B. nana* or *S. pulchra* to show significant changes in production. Short- and long-term responses to warming in the moist acidic tundra have been shown to vary, in part because of slow recruitment and establishment of new individuals (Hollister *et al*. 2005*b*). For example, observations from the ITEX experiments showed that community changes in deciduous shrubs did not become significant until after 4 years of warming (Walker *et al*. 2006). However, since we measured growth at an individual (rather than community) level in order to detect within-season changes of biomass accumulation, the response of deciduous shrubs may be more likely attributed to nutrient availability in that year. If evergreen shrubs were able to access nutrient pulses early in the season before deciduous shrubs, it may help explain why the latter showed little response, specifically when snow was melted early. The one forb tested in this experiment, *P. bistorta*, had highly variable results which may have obscured any treatment effects.

While the magnitude of temporal shifts is often a focus of phenological studies, our results suggest that evolved strategies within the plant community also play an important role in determining responses to altered seasonality. We predicted that earlier leaf expansion would lead to greater production, and we found that this was true for early expanding species but not later expanding species. This demonstrates that temporal niche partitioning influences species’ responses to environmental change. A previous study (Cleland *et al*. 2012) examined plant responses to warming and found that phenologically flexible species (able to ‘track’ climate change) had positive performance responses (e.g. increased abundance and production). Our results are only partially consistent with this result. In our study, changes in phenology alone did not always result in a change in production. Rather, community patterns of leaf expansion, along with warming-driven increases and decreases in ANPP (Fig. 5), contributed to a negative relationship between spring phenology and production (Fig. 4). If this relationship was representative of differences in functional groups alone, we would expect the relationship to hold among control plots, which was not the observed result (Fig. 5). Differences in the ability of species to shift the timing and rate of leaf expansion may affect competitive interactions and subsequently influence future plant community composition (Richardson *et al*. 2010; Cleland *et al*. 2012). Specifically, *E. vaginatum*, which was able to green rapidly and maintain its growth rate, may have a competitive advantage. Further, we predict that species that occupy early-season temporal niches across diverse ecosystems may increase in abundance under altered climate conditions.

## Conclusions

Changes in vegetative phenology, regardless of changes in production, have important implications for functioning of Arctic ecosystems. Phenological shifts can affect competition among species, and differential responses of individual species may determine future plant community structure. Changes in Arctic plant communities have the potential to affect multiple aspects of ecosystem function, including (i) carbon cycling, by altering the balance between ecosystem-scale photosynthesis and respiration (Shaver *et al*. 1992; Hobbie *et al*. 2000); (ii) surface energy balance and feedbacks to the climate system, through change in albedo and seasonal changes in leaf area (Peñuelas *et al*. 2009; Richardson *et al*. 2013); and (iii) trophic relationships that may become decoupled if plant phenology responds to a changing climate differently than vertebrate and invertebrate herbivores (Post and Forchhammer 2008; Høye *et al*. 2013). Our study suggests that an earlier spring as indicated by satellite data may be driven by early greening species such as *E. vaginatum* and *C. bigelowii*. These species have the advantage of being able to respond rapidly and positively to changes in seasonality, and may increase in abundance in tundra ecosystems as earlier snowmelt and warmer springs continue.

## Sources of Funding

Funding for the Snowmelt Project was provided by the National Science Foundation Office of Polar Programs Grants #PLR-1007672, 0902096 and 0902184. Additional funding for C.L. was provided by a National Science Foundation Graduate Research Fellowship.

## Contributions by the Authors

M.N.W., M.W., P.F.S., A.D.-N. and H.S. designed and implemented the experiment. C.L., A.D.-N. and H.S. collected data. C.L. and H.S. analysed the data and wrote the manuscript, and all authors contributed to revisions.

## Conflict of Interest Statement

None declared.

## Supporting Information

The following additional information is available in the online version of this article —

**Table S1.** Species composition at Imnavait Creek. Per cent cover estimates are averaged over subplots for the entire experimental site.

**Table S2.** Microclimate variables in all 3 years of the experiment (2010–12). Air temperature, soil temperature and soil moisture were measured with automated sensors at each subplot throughout spring and summer, and are presented here as means over the observation period ± 1 SEM.

Additional Information
