# Supplementary material for: Earlier snowmelt and warming lead to earlier but not necessarily more plant growth
Source: AoB Plants. 2016 Apr 13;8:plw021. doi: 10.1093/aobpla/plw021 (PMC4866651; doi:10.1093/aobpla/plw021)
Supplement: Additional Information [file supp_8_plw021_index.html]

Earlier snowmelt and warming lead to earlier but not necessarily more plant growth — Earlier snowmelt and warming lead to earlier but not necessarily more plant growth — Additional Information 

# Earlier snowmelt and warming lead to earlier but not necessarily more plant growth

## Additional Information

Additional Information

- Additional Information - Docx file
