# Supplementary material for: Earlier snowmelt and warming lead to earlier but not necessarily more plant growth
Source: AoB Plants. 2016 Apr 13;8:plw021. doi: 10.1093/aobpla/plw021 (PMC4866651; doi:10.1093/aobpla/plw021)
Supplement: Additional Information [file supp_plw021_plw021supp.docx]

**Supplementary Table 1:** **Species composition at Imnavait Creek.** Percent cover estimates are averaged over subplots for the entire experimental site.

| Species | % Cover |
| --- | --- |
| Moss | 52.00 |
| *Betula nana* | 8.14 |
| *Vaccinium vitis-idaea* | 7.06 |
| Lichen | 4.47 |
| *Eriophorum vaginatum* | 4.03 |
| *Salix pulchra* | 3.92 |
| *Ledum palustre* | 3.70 |
| *Carex bigelowii* | 3.12 |
| *Cassiope tetragona* | 2.11 |
| *Polygonum spp.* | 0.96 |
| *Pedicularis oederi* | 0.93 |
| *Petasites frigida* | 0.57 |
| *Salix phlebophylla* | 0.35 |
| *Vaccinium uliginosum* | 0.30 |
| *Pedicularis lapponica* | 0.27 |
| *Rubus chamaemorus* | 0.23 |
| *Pyrola grandiflora* | 0.22 |
| *Hierachum spp.* | 0.10 |
| *Andromeda polifolia* | 0.07 |
| *Empetrum nigrum* | 0.05 |
| *Oxycoccus microcarpus* | 0.03 |
| *Saxifraga punctate* | 0.03 |
| *Calamagrostis lapponica* | 0.02 |
| *Poa arctica* | 0.01 |

**Supplementary Table 2: Microclimate variables in all three years of the experiment (2010-2012).** Air temperature, soil temperature, and soil moisture were measured with automated sensors at each subplot throughout spring and summer, and are presented here as means over the observation period ± 1 s.e.m.

| **2010** | **Control** | **Early Snowmelt** | **Warming** | **Combined** |
| --- | --- | --- | --- | --- |
| **Air Temperature (°C)** | 9.6 ± 0.18 | 9.4 ± 0.17 | 11.3 ± 0.19 | 11.1 ± 0.17 |
| **Soil Temperature (°C)** | 7.3 ± 0.09 | 8 ± 0.08 | 7.7 ± 0.08 | 7.7 ± 0.08 |
| **Soil Moisture (Volumetric Water Content)** | 0.22 ± 0.003 | 0.18 ± 0.003 | 0.19 ± 0.004 | 0.07 ± 0.002 |
| **2011** | | | | |
| **Air Temperature (°C)** | 9.3 ± 0.17 | 9.2 ± 0.16 | 11.2 ± 0.17 | 10.9 ± 0.17 |
| **Soil Temperature (°C)** | 6.8 ± 0.08 | 7.9 ± 0.09 | 7.5 ± 0.08 | 8.2 ± 0.1 |
| **Soil Moisture (Volumetric Water Content)** | 0.18 ± 0.002 | 0.16 ± 0.002 | 0.17 ± 0.001 | 0.13 ± 0.004 |
| **2012** | | | | |
| **Air Temperature (°C)** | 10.9 ± 0.19 | 10.9 ± 0.19 | 12.4 ± 0.21 | 12.2 ± 0.22 |
| **Soil Temperature (°C)** | 8.3 ± 0.13 | 8.8 ± 0.11 | 9.2 ± 0.11 | 9.8 ± 0.14 |
| **Soil Moisture (Volumetric Water Content)** | 0.21 ± 0.005 | 0.16 ± 0.003 | 0.21 ± 0.005 | 0.13 ± 0.002 |
